# Effects of treadmill exercise on cerebral angiogenesis and MT1‐MMP expression after cerebral ischemia in rats

**DOI:** 10.1002/brb3.1079

**Published:** 2018-07-23

**Authors:** Yi Tang, Yixian Zhang, MouWei Zheng, Jianhao Chen, Hongbin Chen, Nan Liu

**Affiliations:** ^1^ Department of Neurology Fujian Medical University Union Hospital Fuzhou China; ^2^ Department of Neurology Fujian Provincial Geriatric Hospital Fuzhou China; ^3^ Department of Rehabilitation Fujian Medical University Union Hospital Fuzhou China; ^4^ Key Laboratory of Brain Aging and Neurodegenerative Diseases Fujian Key Laboratory of Molecular Neurology Fujian Medical University Fuzhou China

**Keywords:** angiogenesis, cerebral ischemia, MT1‐MMP, rehabilitation, treadmill exercise training

## Abstract

**Introduction:**

Despite the increased understanding of treadmill training on angiogenesis of stroke patients, its mechanism is not clearly known. The metalloproteinase membrane type 1‐metalloprotease (MT1‐MMP) promotes the regeneration of the peripheral vessels but seldom research on the regeneration of cerebral blood vessels. This study was designed to investigate the effects of treadmill exercise on angiogenesis and MT1‐MMP expression after cerebral ischemia in rats.

**Methods:**

The adult male Sprague Dawley(SD) rats were randomly divided into three groups: sham operation group, the middle cerebral artery occlusion group(MCAO) and middle cerebral artery occlusion group(MCAO)+exercise group. In 4d, 7d or 14d after MCAO, respectively, the rats’ neurological function was evaluated by the modified neurologic severity scores (mNSS); the microvessel numbers in areas surrounding cerebral ischemia were counted with Microvessel Density(MVD)analysis; the levels of MT1‐MMP and reversion‐inducing cysteine‐rich protein with Kazalmotifs (RECK) were detected by Western‐blot and immunohistochemical method.

**Results:**

Compared with MCAO group, the number of capillaries and the level of MT1‐MMP expression around the area of cerebral ischemia were significantly increased in each exercise group (*p *<* *0.05), while the level of RECK expression and the scores of mNSS in each exercise group were significantly decreased (*p *<* *0.05).

**Conclusion:**

This study suggested that treadmill exercise training can significantly promote angiogenesis and improve neurological function after cerebral ischemia. Its mechanism may be related to the upgraduation of the MT1‐MMP expression in brain microvessels surrounding area of the ischemic rat.

## 1 INTRODUCTION

Rehabilitation training plays an important role in neurological function recovery after ischemic stroke. Many studies have shown (Mikhail & Steinstraesser, 2011) that treadmill exercise is conducive to the rehabilitation of cerebral ischemia. In this process, angiogenesis is important in protecting the neurons, and the embryonic stage is extremely important in angiogenesis.

More and more evidences show that MT1‐MMP take action in the beginning of vascular matrix dissolution, endothelial cell growth, vascular remodeling and ultimately in angiogenesis (Biernaskie, Cheruenko, & Corbett, 2004). Meanwhile, MT1‐MMP plays a critical role in stabling the vessels, reducing leakage, and promoting regeneration after vascular ischemia (Sounni et al., 2010). There are many evidences showing that MT1‐MMP promotes the regeneration of the peripheral vessels (Kwak et al., 2012) but seldom research on the regeneration of cerebral blood vessels.

In this study, we established a MCAO model, and detected the vascular regeneration and the expression of MT1‐MMP before and after treadmill exercise in rat brain tissue, so as to provide some theoretical basis for the rehabilitation training in ischemic stroke. We found that treadmill exercise induces angiogenesis of brain. At the same time, MT1‐MMP positive expression was visible on microvascular endothelial cells marked by FVIII–R Ag, which demonstrated that MT1‐MMP has a role in angiogenesis around ischemic area. On the other hand, reversion‐inducing cysteine‐rich protein with Kazalmotifs (RECK) is one of the inhibitor of MT1‐MMP in other models (Silva et al., 2015; Oh et al., 2001), but whether RECK suppress the function of MT1‐MMP in stroke is not clear. This study suggested that treadmill exercise can promote angiogenesis, up‐regulate the expression of MT1‐MMP and down‐regulate the expression of RECK on vascular endothelial cells around cerebral ischemic area.

## 2 MATERIAL AND METHODS

### 2.1 Experimental animals and grouping

The adult male SD rats, weighing 250‐280 g, were supplied by Hayes Reich company,(license number: SCXK Shanghai 2007‐0005).All rats were housed in groups of two and maintained under a 12‐hr light–dark cycle with food and water available ad libitum. All experimental procedures were performed during the light phase. Rats were randomly divided into three groups: sham group, MCAO group, treadmill exercise group. Exclude sham group, each group was divided into three subgroups (*n* = 6 for each subgroup); each group was further divided into three groups: 4d, 7d, and 14d after MCAO, with six rats in each subgroup. Measurement of motor function, analysis of angiogenesis and MT1‐MMP were performed in each subgroup. The experimental animal disposal process is in accordance with the relevant standards formulated by the animal ethics Committee of Fujian Medical University.

### 2.2 Treadmill exercise

All rats underwent a 3‐day adaptive run‐training on a treadmill before surgery. The treadmill exercise group was trained 24 hr after surgery until a predetermined time point. The exercise strength grew gradually, with the parameters in briefly. The rats began training at a speed of 4 m/min per day for 2 days, then 8 m/min on the third day. Then the speed increased to 12 m/min for 30 min daily for 5 days a week, which lasted for 2 weeks.

### 2.3 Neurological deficits

Using the modified neurological severity scores (mNSS) method, MCAO rats in each group were evaluated on 4d, 7d, and 14d after MCAO, respectively. The mNSS includes motor, sensory, reflex, and balance, total score 18 points. A higher score means the more severe neurological deficits. The score for a normal rat is 0 point.

### 2.4 Preparation of models

All animals used in this study were cared for in accordance with the National Institute of Health Guide for the Care and Use of Laboratory Animals (NIH Publications No. 80‐23, revised in 1996). All procedures were approved by Institutional Animal Care and Use Committee of Fujian Medical University. Efforts were made to minimize the number of animals used as well as their suffering. Referring to the modified Zea‐Longa suture method (Galvez et al., 2005), we prepared MCAO rat models.

### 2.5 Morphometric analysis of cerebral infarcts

To determine infarct volume, rats were sacrificed 7 Days after surgery and the brain was sliced into 2‐mm coronal sections. Sections were stained with 2, 3, 5‐triphenyltetrazolium chloride (TTC). Infarcted areas were analyzed using ImageJ.

### 2.6 Immunohistochemistry

The cerebral hemisphere was extracted after transcardial perfusion with 4% paraformaldehyde (solution in 0.1 mol/L phosphate buffer, pH = 7.4). The sample was then dehydrated in 20% sucrose solution in 4% paraformaldehyde, followed by 30% sucrose solution in 0.1 mol/L phosphate buffer. Nonspecific blocking was performed on the sections of 1 hr with 10% goat serum at room temperature. Incubation with polyclonal rabbit anti‐MT1‐MMP(1: 250, abcam) and a polyclonal mouse anti‐RECK (H‐300)(1:50, santa) antibodies subsequently performed at room temperature for 1 hr and then overnight at 4°C. The sections were then incubated in two fluorescence coupled secondary antibodies (Cy3 goat anti‐rabbit IgG (H + L), 1:300, Beyotime; FITC goat anti‐mouse IgG (H + L), 1:400, Beyotime) for 1 hr at room temperature. Three sections (Bregma 1.0 mm) obtained from each animal were selected for analysis. Three randomly selected areas in the ischemic penumbra of each section were digitized into TIFF images using the same exposure time. Image analysis was as follows: 10 high‐power fields were randomly selected from each slice; the corresponding fields selected from each slice were of the same site; the number of positive cells in the immune response were counted; MVD count followed the method of (Weidner, Semple, Welch, & Folkman, 1991), then image transferred to a 200 times fluorescence microscope to count the microvessels; the average number was taken as the microvessel density.

### 2.7 Western blotting

After a predefined period of 1 or 2 weeks after the ischemia procedure, the rats were killed under ketamine anesthesia by intracardiac perfusion with 200 ml of 0.9% NaCl. Concentration of the protein was measured by the BCA protein assay (Beyotime, China). Extracts were separated by 10% or 12% sodium dodecyl sulfate‐polyacrylamide gel electrophoresis (SDS‐PAGE) and transferred to PVDF membranes (Millipore, USA). Membranes were probed with primary antibodies at 4°C overnight (rabbit anti‐MT1‐MMP, abcom, 1:1,000; mouse anti‐RECK (H‐300), santa company, 1:200; goat anti‐rabbit IgG (H + L), l:2,000; goat anti‐mouse IgG (H + L), l:3,000). Band intensities were analyzed with the image J software (1.46r).

### 2.8 Statistical methods

All of the experimental data were determined by homogeneity test of variance and normality test, expressed as Mean ± *SD* and analyzed by SPSS 13.0 software; pairwise comparison between groups was tested by SNK; *p *<* *0.05 indicated statistical significance.

## 3 RESULTS

### 3.1 mNSS nerve function score

**Figure 1:**
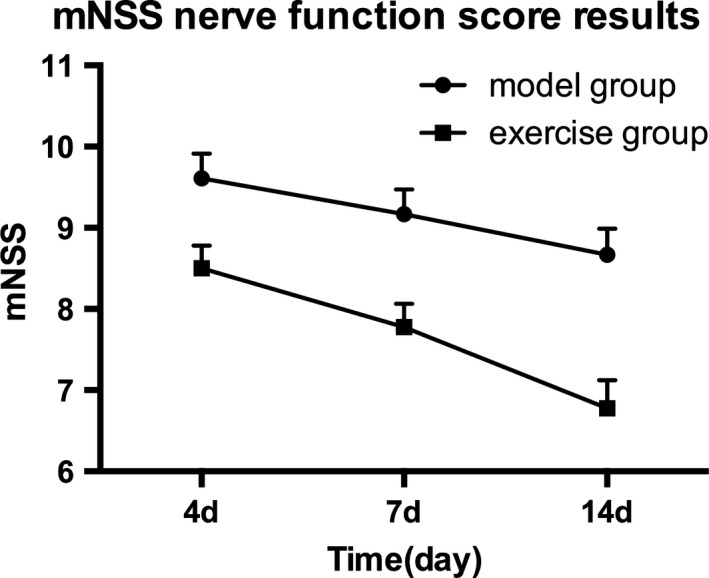
mNSS Nerve function score. The exercise group compared with the model group, *p < 0.05

4d after MCAO, compared with the model group, the exercise group showed no statistical significance in mNSS nerve function scores (*p* > 0.05); 7d and 14d after MCAO, the exercise group got better function scores than the model group in the same period (*p* < 0.05). (Figure 1).

### 3.2 TTC analysis results

**Figure 2:**
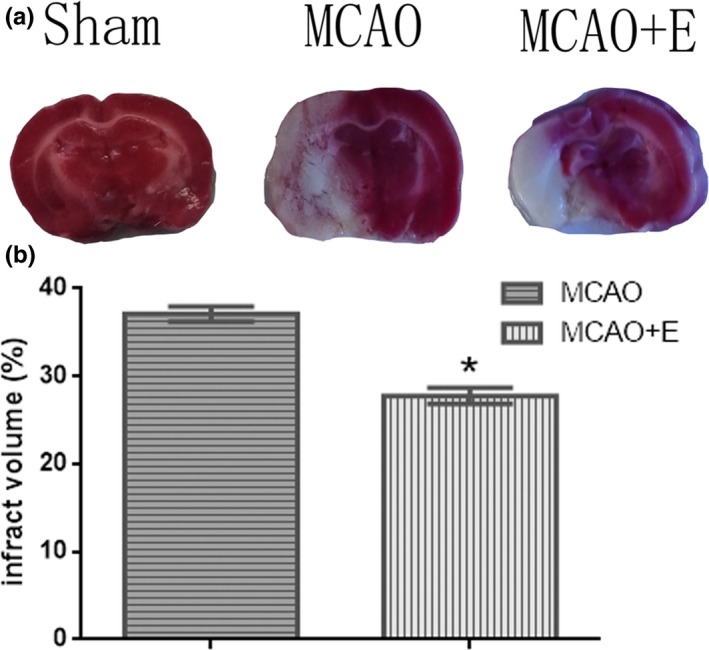
Treadmill exercise reduces infarct volume after middle cerebral artery occlusion (MCAO). (a) Representative coronal brain sections stained with 2, 3, 5‐triphenyltetrazolium chloride. (b) Quantitative analysis of infarct volumes. *p < 0.01 (*n* = 3 in each group)

Compared with sham and MCAO group, the area of infarction in the exercise group decreased significantly, and the difference was statistically significant (*p *<* *0.05, Figure 2).

### 3.3 Analysis of Microvessel Density (MVD) in ischemic region

**Figure 3:**
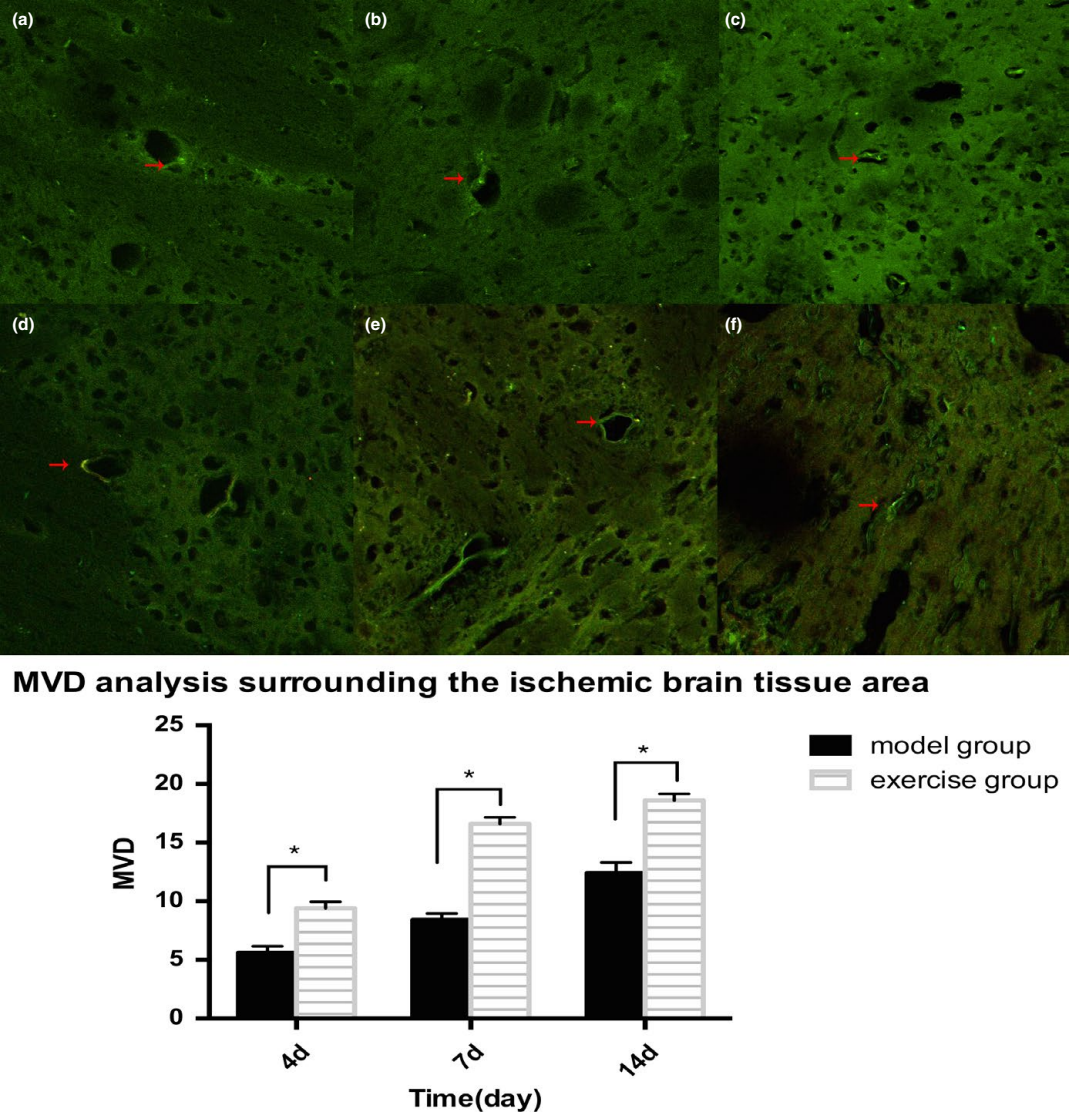
The vessels marked by FVIII‐R Ag could be seen scattered around the ischemic rat brain in model and exercise group. a: 4d, b:7d, c:14d, in model group microvessel density peaked on 14d. (Scale = 100um).d:4d,e:7d,f:14d,in exercise group microvessel density compared with the model group(at 4d,7d,14d), *p < 0.05

Through the laser scanning confocal microscope, the endothelial cells marked by FVIII‐R Ag could be seen scattered around the ischemic rat brain after successful MCAO. The calculation of microvessel density showed that: On 4d, the newborn capillaries were visibly surrounding the ischemic rat brain in MCAO group; the number of newborn microvessels gradually increased and peaked during 7‐14d; at the corresponding time points, the exercise group showed a more evident neovascularization phenomenon, and was statistically significant compared with MCAO group (*p *<* *0.05, Figure 3).

### 3.4 Protein expression of MT1‐MMP and RECK around ischemic brain

**Figure 4:**
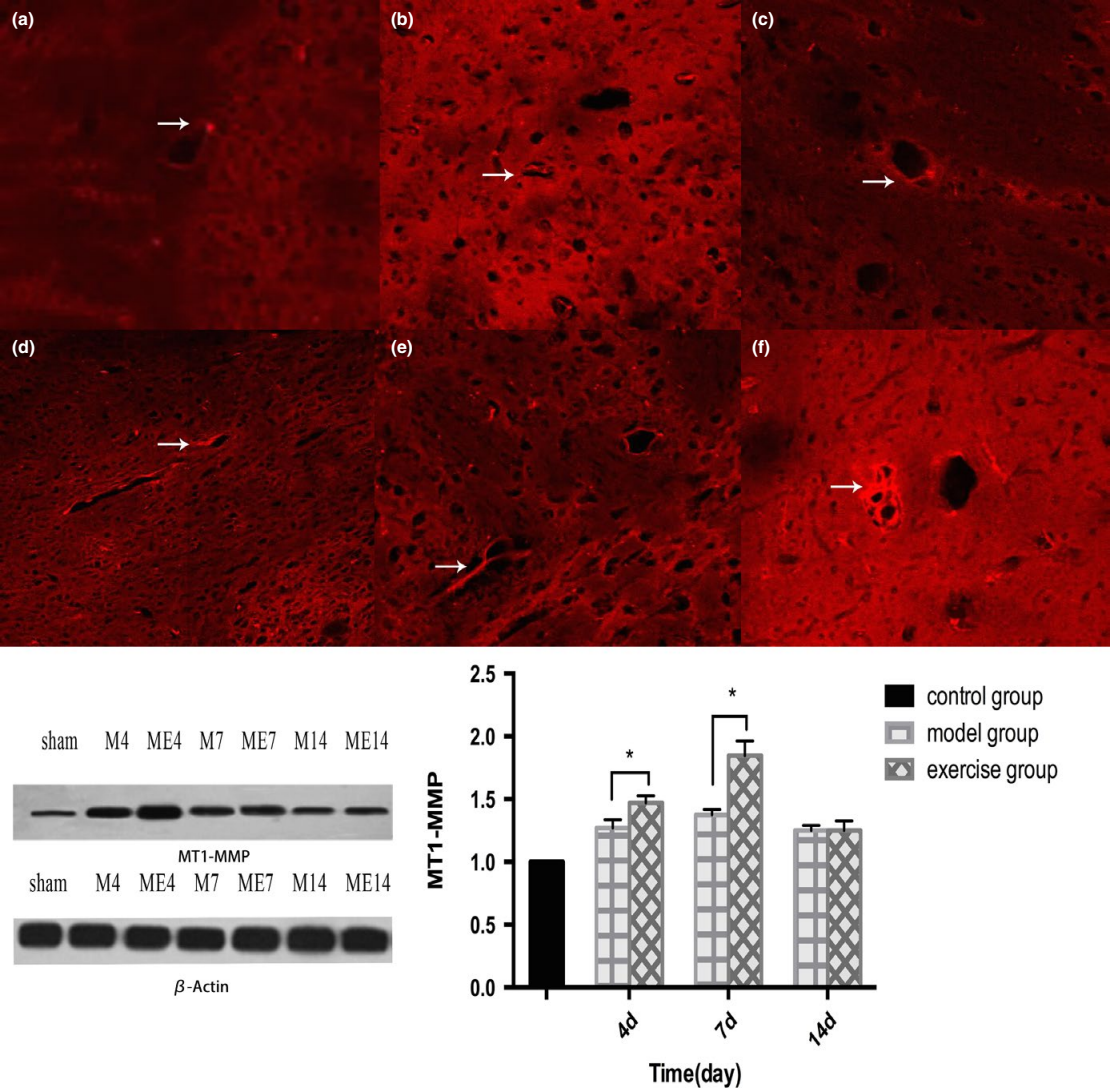
The expression of MT1‐MMP in rat brain after middle cerebral artery occlusion (MCAO)(a:4d,b:7d,c:14d)and treadmill exercise (d:4d,E:7d,14d). The exercise group compared with the model group, *p < 0.05

1. Sham group showed low levels of MT1‐MMP expression. In model group, the protein expression levels of MT1‐MMP increased on 4d postoperative and peaked on 7d, but decreased on 14d. In exercise group, the expression levels also increased on 4d postoperative, peaked on 7d, and decreased on 14d. On 4d and 7d, the expression of the exercise group were significantly higher than those of MCAO group, and the difference was statistically significant (*p* < 0.05, Figure 4); on 14d, compared with the model group, the expression levels were mildly elevated in exercise group, but the difference was not statistically significant (*p* > 0.05, Figure 5).

**Figure 5:**
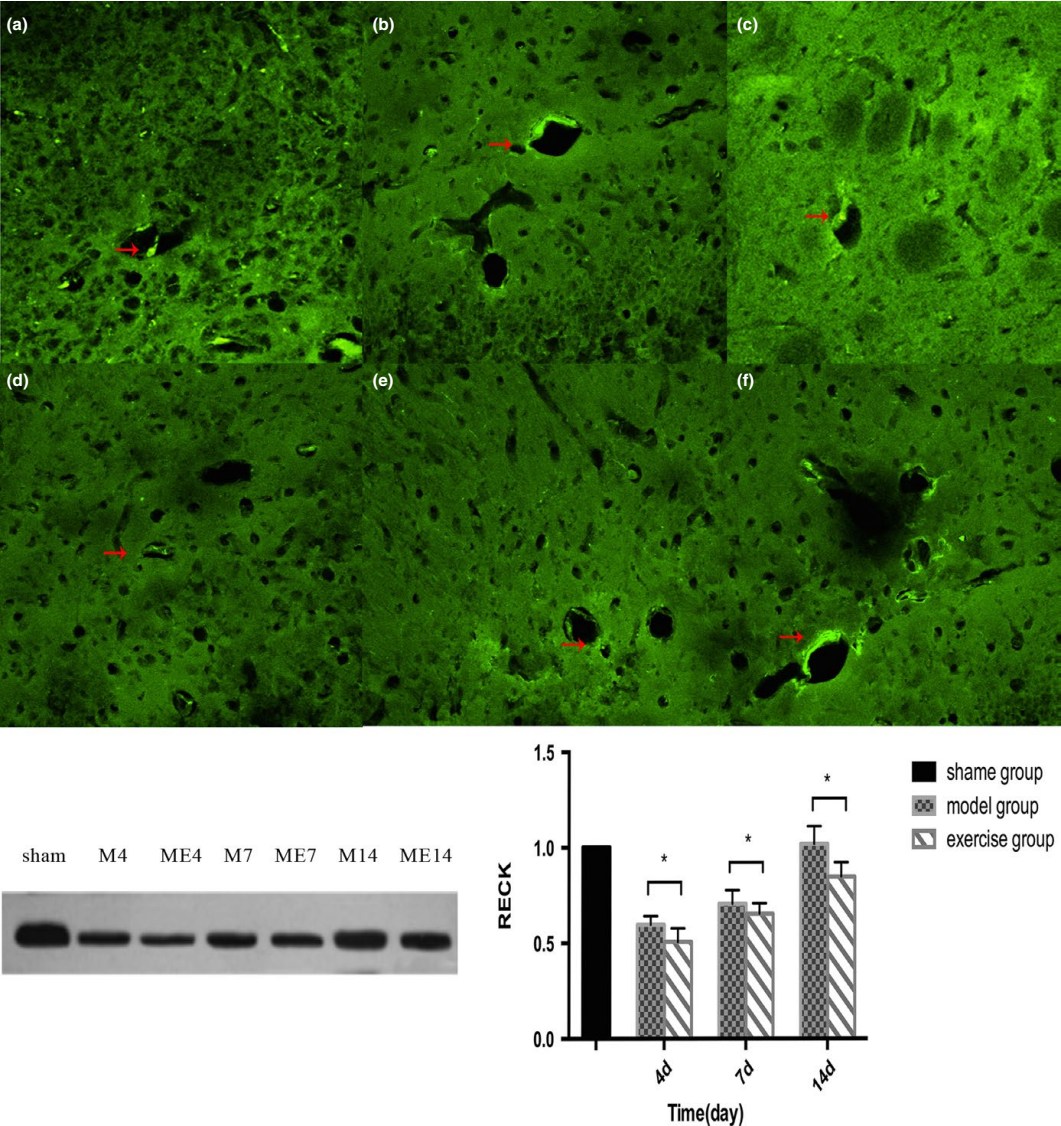
The expression of reversion‐inducing cysteine‐rich protein with Kazalmotifs (RECK) in rat brain after middle cerebral artery occlusion (MCAO) (a:4d, b:7d, c:14d)and treadmill exercise (d:4d, e:7d, f:14d). The exercise group compared with model group(at 4d,7d,14d), *p < 0.05
2. Reversion‐inducing cysteine‐rich protein with Kazalmotifs expression in the Sham group was of a higher level. In the model group, RECK protein expression reached the lowest level on 4d postoperative, and somewhat restored on 7d and 14d. In exercise group, the expression levels also declined on 4d after surgery, and recovered on 7d and 14d. On 4d and 7d, compared with those in MCAO group, RECK expression levels in exercise group decreased, and the difference was statistically significant (*p* < 0.05, Figure 6).

**Figure 6:**
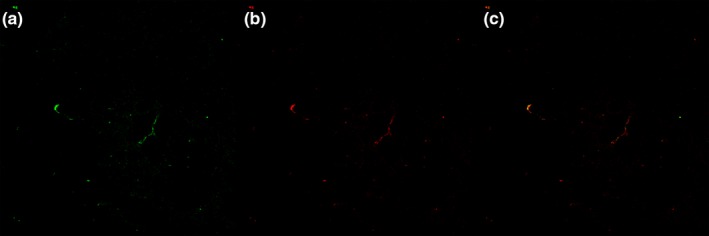
Co‐expression of MT1‐MMP receptor and FVIII‐R Ag‐marked endothelial cells on microvascular in angiogenesis
3. MT1‐MMP and FVIII‐R Ag were co‐expressed in the Microvascular endothelial cells of the peripheral zone of cerebral ischemia, suggesting that MT1‐MMP was involved in the hyperplasia of microvessels in the peripheral zone of ischemia.

## 4 DISCUSSION

Cells on the verge of apoptosis can be saved by improving cerebral blood flow after the ischemia focus and promoting angiogenesis and vascular remodeling in the early stage of the ischemic cerebrovascular disease. Therefore, it is important in the clinical treatment of cerebral ischemia to improve or restore cerebral blood flow in early cerebral ischemia. Many evidences have shown that a higher microvessel density means a significantly better prognosis for patients with stroke, and angiogenesis can improve blood perfusion in brain tissue surrounding the ischemic area in rats and promote neurological function recovery after ischemia (Zacharek et al., 2007). Meanwhile, some studies found that cerebral ischemia can cause auto‐angiogenesis in the ischemic region (Beck & Plate, 2009). Angiogenesis improves blood perfusion in the ischemic penumbra region, thus diluting or even removing the necrotic tissue and damaging inflammatory substances in ischemic area, which helps to stabilize neovascularization, and ultimately promotes the neurological function recovery after cerebral ischemia (Arenillas, Sobrino, Castillo, & Dávalos, 2007). Expression of a variety of pro‐angiogenic cytokines, such as vascular endothelial growth factor, increase in the brain after ischemia, and the corresponding receptor up‐regulate; these factors induce proliferation and migration of vascular endothelial cells, thereby promote angiogenesis (Brea, Sobrino, Ramos‐Cabrer, & Castillo, 2009). However, these blood vessels developed in auto‐angiogenesis cannot fully meet the needs of neurological recovery.

The present study demonstrated that treadmill exercise can induce angiogenesis in striatal and other parts of cerebral and improve balance and coordination in MCAO rats. Improvement in motor function was associated with striatal angiogenesis after exercise. In addition, treadmill exercise at both speeds reduced infarct volume in MCAO rats. So the main object of ischemic stroke rehabilitation is the enhancement of brain vascular hyperplasia in the ischemic area by treadmill exercise (Yang, Chang, Wang, & Wang, 2012).

The reason why treadmill exercise can promote angiogenesis and rebuild collateral circulation may be related to that treadmill exercise increases brain angiogenic factors, matrix metalloproteinases‐2, and MT1‐MMP (He et al., 2014). MT1‐MMP can alter the environment among cells, leading to changes of vascular endothelial cell adhesion molecules or signal transduction, thereby affecting the function of cells, including endothelial cells, and then promote angiogenesis. In the process of angiogenesis, the growth of the embryonic stage is extremely important; and studies have shown that MT1‐MMP plays an extremely important role in endothelial cell growth, sprouting angiogenesis and vascular remodeling (Ohkawara et al., 2014).

Reversion‐inducing cysteine‐rich protein with Kazalmotifs genes are divided into two kinds in vivo, that is, membrane RECK and soluble RECK (Cho, Smallwood, & Nathans, 2017); bothcan be weakly expressed in normal tissues. Studies have shown that post‐transcriptional RECK can inhibit MT1‐MMP. RECK enables tissues to strengthen remodeling and repairing by maintaining blood vessels and nerve tissue around ECM, which is very important during embryonic development (Liu, Zhou, & Zhu, 2016; Oh et al., 2001). Studies about mouse embryos showed that knocking out RECK gene will lead to death of the mouse embryos at early stages. Anti‐miRs increased expression of RECK mRNA and protein in HT1080 fibrosarcoma cells, but, decreased RECK mRNA and increased its protein in the benign prostatic hyperplasia cell line BPH‐1. Treatment of BPH‐1 and HT‐1,080 cells with the anti‐miRs did not change the level of cell surface MT1‐MMP activity. Trichostatin A (TSA) did not increase the level of RECK, but blocked cell surface MT1‐MMP activity and decreased cell motility. Anti‐miRs mediated increased RECK levels did not interfere with cell surface MT1‐MMP function, and TSA may block cell surface localization of MT1‐MMP by a mechanism independent of RECK. In different cell lines, RECK may play different roles in regulation of MT1‐MMP (Silva et al., 2015. The activity of RECK and MT1‐MMP is essential for angiogenesis (Miki et al., 2010; Sandri et al., 2016). The increased RECK expression will inhibit angiogenesis, and vice versa (Golan, Vagima, & Goichberg, 2011).

In this experiment, the MCAO rat model was established successfully, and the exercise group was trained on treadmill. Compared with the MCAO group, the area of infarction in the exercise group was reduced and the score of nerve function was improved. Large numbers of neovascularization could be seen around ischemic cerebral areas after treadmill exercise. Compared with the MCAO group, the increasing of angiogenesis in exercise group was dramatic, which confirms that treadmill exercise can promote angiogenesis. We found that MT1‐MMP positive expression was visible on microvascular endothelial cells marked by FVIII–R Ag, which demonstrated that MT1‐MMP has a role in angiogenesis around ischemic area. Moreover, we found that MT1‐MMP protein expression began to rise on 4d after ischemia, and peaked on 7d; MT1‐MMP protein levels in exercise group was significantly higher than those in MCAO group on 4d, 7d after ischemia, which suggested that treadmill exercise could promote MT1‐MMP expression around cerebral ischemic area; MT1‐MMP protein levels increased not significantly on 14d after ischemia, indicating that MT1‐MMP plays a major role in early angiogenesis, but not in the latter stages. However, the results showed that the expression and change trend of RECK was contrary to MT1‐MMP. The research showed that MT1‐MMP expresses mainly in fibroblasts, smooth muscle cells, and endothelial cells. Gel zymography showed that treadmill exercise can suppress the ability of MT1‐MMP to activate pro‐MMP2 to MMP2 (Al‐Raawi, Abu‐El‐Zahab, El‐Shinawi, & Mohamed, 2011). However, epithelial cells do not express activated MT1‐MMP in general. When the tissue is damaged, such as cerebral ischemia, expression and activation of MT1‐MMP starts and MT1‐MMP breakdown I, II, and III collagens, fibronectin, laminin‐1, 5, and other ECM macromolecules (Shi & Sottile, 2011). Therefore, the expression and activation of MT1‐MMP promotes angiogenesis in the early ischemic stroke; However, the results also showed that the expression and change trend of RECK was contrary to MT1‐MMP, which means RECK may be a potential inhibitor of MT1‐MMP after stroke. Thus, treadmill exercise protects the brain partly by increasing the expression of MT1‐MMP and promoting angiogenesis, especially in the early stages.

This study suggested that treadmill exercise can promote angiogenesis and the expression of MT1‐MMP on vascular endothelial cells around cerebral ischemic area. Treadmill exercise promotes angiogenesis probably by up‐regulating the expression of MT1‐MMP, thus protecting the brain against cerebral ischemia in rats.

## CONFLICT OF INTEREST

The authors declare that they have no conflict of interests.
